# Gum–Gelatin Nanocapsules of Pomegranate Phenolic Extract Promote Redox Homeostasis, Metabolic Health, Immunity, Gut Microbiota, and Growth in Newly Weaned Rabbits

**DOI:** 10.3390/ani16010069

**Published:** 2025-12-26

**Authors:** Nesrein M. Hashem, Nourhan S. Hosny, Nagwa El-Desoky, Sanaa S. Elalfy, Mohamed S. Mohamed, Ali A. El-Raghi, Zahraa R. Abo-Elezz

**Affiliations:** 1 Animal and Fish Production Department, Faculty of Agriculture (El-Shatby), Alexandria University, Alexandria 21545, Egypt; enagwa278@gmail.com (N.E.-D.); mkosba@hotmail.com (Z.R.A.-E.); 2 Livestock Research Department, Arid Lands Cultivation Research Institute, City of Scientific Research and Technological Applications (SRTA-City), Alexandria 21934, Egypt; nourhansaad80@yahoo.com; 3 Animal and Poultry Production Department, Faculty of Agriculture, Damanhour University, Damanhour 22514, Egypt; sanaa.salama@agr.dmu.edu.eg (S.S.E.); m.sami.elsanm@gmail.com (M.S.M.); 4 Animal, Poultry, and Fish Production Department, Faculty of Agriculture, Damietta University, Damietta 34517, Egypt; ali21384@yahoo.com

**Keywords:** nanoencapsulation, pomegranate peel extract, rabbit growth, gut microbiota, antioxidant status

## Abstract

Pomegranate peel, a rich agro-industrial by-product, contains abundant phenolic compounds with strong antioxidant and antimicrobial properties. However, the low stability and bioavailability of these compounds limit their efficacy in animal nutrition. This study investigated the effects of pomegranate peel phenolic extract (PE), either in raw form (PE300) or nano-encapsulated using gum–gelatin nano-capsules (NPE300), on health and growth parameters in newly weaned rabbits. Fifty-four male rabbits (40 days old) were assigned to three treatment groups: PE0 (control), PE300 (300 mg PE/L drinking water), and NPE300 (300 mg nano-encapsulated PE/L drinking water). Over six weeks, growth performance, hematological and immunological profiles, antioxidant status, microbial populations, and carcass traits were evaluated. NPE300 treatment demonstrated superior antimicrobial activity *in vitro*, with larger inhibition zones against all tested pathogens compared to PE300. *In vivo*, NPE300 significantly improved body weight gain (945.8 g) and feed efficiency, while also enhancing immune function, evidenced by higher white and red blood cell counts, phagocytic activity, and increased plasma IgG and IgM levels. Antioxidant markers showed that NPE300 significantly reduced malondialdehyde levels and tended to improve total antioxidant capacity. Furthermore, intestinal *Clostridia* counts were reduced, and beneficial microflora significantly increased in the NPE300 group. Carcass weight with edible parts, fur weight, kidney weight, and cecum length were also elevated under NPE300 treatment. In conclusion, nanoencapsulation of PE using gum–gelatin carriers enhanced its bio-efficacy, supporting better redox balance, immunity, gut health, and growth performance in rabbits. These findings support the application of nano-encapsulated PE as a promising natural growth promoter in rabbit production.

## Simple Summary

Ensuring the production of safe and sustainable animal products is an urgent global priority, particularly as antimicrobial resistance continues to pose serious health risks to both humans and animals. Developing natural alternatives with multiple biological activities offers a potentially effective and environmentally friendly strategy for animal production systems, helping to improve health and overall well-being. Enhancing the efficacy of phytogenic compounds through nanoencapsulation has emerged as a promising approach to support their stability, bioavailability, and biological potential. In this context, the present study investigated the effects of pomegranate peel phenolic extract (PE) on the health and performance of newly weaned rabbits. Fifty-four male rabbits, aged 40 days, were randomly assigned to three treatment groups: PE0 (control), PE300 (300 mg PE/L of drinking water), and NPE300 (300 mg nanoencapsulated PE/L). Results demonstrated that nanoencapsulation of PE using gum–gelatin carriers significantly enhanced its bioefficacy, improving redox balance, immune responses, gut health, and growth performance in weaned rabbits.

## 1. Introduction

Weaning is one of the most critical and stressful phases in rabbit production, often associated with marked physiological, metabolic, and immunological changes [1]. Newly weaned rabbits face significant challenges due to abrupt dietary transitions and immature digestive and immune systems, which can lead to oxidative stress, impaired growth performance, intestinal dysbiosis, and increased susceptibility to infections [2]. These stressors collectively threaten rabbits’ welfare and productivity, underscoring the need for effective nutritional interventions to support redox balance, immunity, and gut health during this vulnerable stage [3]. Among the promising natural solutions, pomegranate peel extract (PE) has gained considerable attention due to its richness in phenolic compounds, which possess potent antioxidant, antimicrobial, and immunomodulatory properties [4,5]. The bioactive compounds in PE can help alleviate oxidative damage, modulate immune responses, and support intestinal microbial homeostasis, thereby improving animal health and performance. As such, PE represents a potential functional feed additive for mitigating weaning-related stress in rabbits [5,6]. Numerous studies have demonstrated the beneficial effects of pomegranate and its byproducts on enhancing growth performance in various farm animals. For instance, incorporating pomegranate byproducts into rabbit diets at levels of 100, 150, and 200 mg/kg has been shown to improve growth rates, reduce pathogenic bacterial populations, and provide a natural source of antioxidants [7]. Additionally, dietary supplementation with pomegranate peel (20 g pomegranate peel per kg of diet) has been effective in mitigating the adverse effects of heat stress. This intervention positively influenced the animals’ nutritional and physiological profiles, leading to improved performance, improved cecal fermentation, and increased antioxidant capacity. Consequently, these benefits contribute to better overall health and productivity in rabbits [8]. However, the application of PE in animal nutrition faces several limitations. These include the inherent instability of phenolic compounds under gastrointestinal conditions, poor solubility, and low bioavailability. Such constraints diminish their overall efficacy, as many active constituents may degrade or be absorbed inefficiently before they can exert their intended health benefits [9,10]. To address these challenges, nanoencapsulation technology has emerged as a promising delivery approach [11]. By encapsulating bioactive compounds within nanoscale carriers—such as gum–gelatin-based nano-encapsules—this technique can significantly improve the stability, solubility, and absorption of phenolic compounds. Nanoencapsulation not only protects these compounds from degradation during digestion but also facilitates targeted release and sustained biological activity, maximizing their therapeutic potential [5]. Moreover, gum–gelatin-based nano-encapsules are biocompatible and non-toxic, making them particularly suitable for inclusion in animal feed formulations [12].

Considering these advancements, the present study aims to evaluate the effects of both raw and nano-encapsulated PE on various physiological and productive parameters in newly weaned rabbits. Specifically, the research will assess the impact on redox balance, immune response, metabolic profiles, intestinal microbial composition, and growth performance indicators.

## 2. Materials and Methods

### 2.1. Fabrication of Pomegranate-Gum Nano-Capsules

The preparation of PE, either as a raw extract or in nanoencapsulated form, was conducted at the Nanoencapsulation and Biotechnology Laboratory (NBL), Faculty of Agriculture, Alexandria University, Egypt. Briefly, dried pomegranate peels were finely ground and sieved through a 1 mm mesh prior to extraction. The resulting powder (15 g/100 mL) was extracted using a 70% (*v*/*v*) hydroethanolic solution at 45 °C for 72 h. The mixture was then filtered through Whatman No. 1 filter paper (Camlab, Cambridge, UK), and the filtrate was stored at 5 °C until further processing [5]. The PE was subsequently lyophilized and used to formulate a nanocomplex of gum Arabic and gelatin (1:1 ratio) Via the ionic-gelation method. For this, 1.5 g of PE (dry matter), containing 300 mg of extract, was dissolved in 15 mL of distilled water and blended with Tween 80 (1% *v*/*v*). This mixture was added to 100 mL of gum Arabic solution (2% *w*/*v*) under gentle magnetic stirring. The resulting blend was then introduced dropwise using a syringe into 100 mL of gelatin solution (2% *w*/*v*), with continuous stirring to promote nano-capsule formation. The resulting nanoparticles were collected by centrifugation and resuspended in water at the desired dose immediately prior to use [13].

### 2.2. Antimicrobial Activities of Pomegranate Phenolic Extract In Vitro

The antimicrobial efficacy of PE formulation was evaluated against six pathogenic microorganisms by measuring the diameters of the inhibition zones using the agar-well diffusion method, as described by Hashem et al. [14]. Microbial isolates used in the study included *Candida albicans* ATCC 10231 (purchased from Becton Dickinson, Le Pont-de-Claix, France), *Escherichia coli* ATCC 25922, *Salmonella typhi* ATCC 19430 (both obtained from the American Type Culture Collection, ATCC, Manassas, VA, USA), *Vibrio cholerae* NCTC 80211, *Pseudomonas aeruginosa* (ATCC 27853) and *Bacillus subtills* NCTC 80211. Additional microbial strains were provided by the Department of Botany and Microbiology, Faculty of Science, Al-Azhar University (Assiut Branch), and the Al-Azhar University Mycology Center (Cairo, Egypt).

### 2.3. Animal Husbandry and Experimental Design

This part of the study was conducted at the Laboratory of Rabbit Physiology Research, Agricultural Experimental Station, Alexandria University, Egypt. The study followed the guidelines of the Pharmaceutical and Fermentation Industries Development Center (PFIDC), General Authority of the City of Scientific Research and Technological Applications (SRTA-City), Egypt. The experimental protocol and procedures were reviewed and approved by the Institutional Animal Care and Use Committee (IACUC) of SRTA-City, under registration number: IACUC # 86-IC-0723. Under standardized management and hygiene conditions, fifty-four male V-line rabbits (40 days old), with an average body weight of 896 ± 20.14 g, were used in a six-week feeding trial. The rabbits were randomly assigned to three treatment groups (18 rabbits per group, six replicates per group) and received either 0 mg/L of pomegranate peel extract in drinking water (PE0, control), 300 mg/L of PE (PE300), or 300 mg/L of nano-encapsulated PE (NPE300). The pomegranate peel extract (PE) was administered at a dose of 300 mg/L in the drinking water of newly weaned rabbits. This dose was selected based on preliminary trials, indicating that this level is safe and effective for promoting growth and health in rabbits, while also allowing evaluation of the potential enhanced effects of the nano-encapsulated form. All animals were individually housed in standard galvanized wire cages (40 × 50 × 35 cm) equipped with automatic feeders and drinkers. Throughout the experiment (conducted in March and April), the average indoor temperature, relative humidity, and photoperiod were 23.14 ± 1.27 °C, 52.47 ± 9.15%, and 11.78 ± 0.74 h, respectively. Rabbits were fed a pelleted diet formulated to meet their daily nutritional requirements [15], consisting of (g/kg diet): 280 alfalfa hay, 250 wheat bran, 180 barley, 180 soybean meal, 60 yellow corn, 30 molasses, 10 calcium carbonate (CaCO_3_), and 10 sodium chloride (NaCl), providing 17.90% crude protein and 10.05 MJ/kg digestible energy.

### 2.4. Growth Performance Indicators

The live body weight (LBW) was recorded weekly throughout the experimental period using a relevance table scale (Honder Weighing Scale Co., Ltd., Taipei City, Taiwan). Body weight gain (BWG) was determined as the difference between the final and initial body weights (BWG = Final body weight − Initial body weight). Feed intake (FI), expressed in grams per rabbit, was measured weekly by weighing the feed offered and subtracting the uneaten residue. The feed conversion ratio (FCR) was calculated as the amount of feed consumed per unit of body weight gain (FCR = g feed/g gain) [16].

### 2.5. Blood Plasma, Biochemical Attributes, Antioxidant Indicators, and Immunological Variables

At the end of the experiment, to assess hematological, biochemical, immunological, and antioxidant parameters, blood samples were collected from the marginal ear vein into heparinized tubes of eight randomly selected rabbits per treatment group. All animals were fasted for 12 h prior to sampling to minimize dietary effects. Hematological parameters—such as red blood cell count, packed cell volume, total white blood cell count with differential, leukocyte counts, and phagocytic activity—were determined. Hemoglobin was measured colorimetrically using commercial kits (Biosystems S.A., Girona, Spain) following the method described by Feldman et al. [17]. Plasma biochemical markers, including total protein, albumin, and glucose, were analyzed using commercial diagnostic kits (Biodiagnostic, Giza, Egypt). Globulin levels were derived by subtracting albumin from total protein concentrations. Triglyceride levels were also determined using commercial kits from the same supplier. Immunoglobulin levels (IgG, IgM, and IgA) were quantified using ELISA kits (IBL America, Minneapolis, MN, USA), with reported sensitivity and specificity greater than 96% [18]. Antioxidant activity was assessed by measuring total antioxidant capacity (TAC) and malondialdehyde (MDA) levels using commercial kits (Biodiagnostic, Giza, Egypt), with detection limits up to 2 mM/L for TAC and 100 nmol/mL for MDA.

### 2.6. Slaughtering and Carcass Characteristics

At the end of the trial, eight rabbits were randomly selected from each experimental group and were humanely sacrificed [14]. The selected animals were fasted for 12 h, individually weighed, and then slaughtered immediately. Slaughtering was carried out following Islamic practices, which involved the use of a sharp knife by a trained individual and the recitation of the appropriate religious phrase, in accordance with the procedure described by Bouzraa et al. [19]. After complete bleeding, the viscera, tail, and pelt were removed. The carcass and all edible parts were weighed, including the edible organs such as the kidneys, heart, lungs, spleen, and the intestinal tract. Measurements were also taken for the lengths of the cecum (in centimeters).

### 2.7. Intestinal Microbial Count Evaluation

The small intestine and cecum were carefully dissected, and their lengths were measured. Contents from the small intestine were individually collected, and samples were obtained for the identification and quantification of specific bacterial populations. This was conducted using media tailored for each bacterial group, including Luria-Bertani broth agar for general microbiota, MacConkey agar for *E. coli*, *Salmonella–Shigella* agar for *Salmonella*, and iron sulfide agar for *Clostridium* detection, following the procedure previously described by Hashem et al. [14] and Cotozzolo et al. [20].

For each bacterial analysis, 5 samples were collected, and each sample was analyzed in triplicate (3 replicates) to ensure accuracy and reproducibility. All media were prepared according to the manufacturer’s instructions, and standard catalog numbers were used as follows: LB agar (Merck, Darmstadt, Germany, Cat. No. 105233), MacConkey agar (Merck, Cat. No. 105464), Salmonella–Shigella agar (Merck, Cat. No. 105368), and iron sulfide agar (Merck, Cat. No. 107333). The bacterial counts were then determined, and data were expressed as colony-forming units (CFU) per gram of intestinal content.

### 2.8. Statistical Analysis

Data analysis was conducted using IBM SPSS Statistics for Windows, version 22.0 (IBM Corp., 2013, Armonk, NY, USA). Hematological, immunological, and biochemical data were analyzed using a Generalized Linear Model (GLM), considering treatment as a fixed effect (inter-subject effect) and repeated measurements within the same animal as a random effect (intra-subject effect). Differences among treatment means were assessed using Tukey’s post hoc test. Results are presented as mean values ± standard error (SE), and statistical significance was considered at *p* < 0.05.

## 3. Results

### 3.1. In Vitro Antimicrobial Activity of Administered Pomegranate-Gum Nano-Capsules

As presented in Table 1, both PE300 and NPE300 treatments significantly increased the inhibition zones against all tested pathogenic microorganisms compared to the control (PE0). The NPE300 treatment consistently produced larger inhibition zones than PE300 across all microorganisms, indicating stronger antimicrobial activity.

### 3.2. Growth Performance

As shown in Table 2, initial body weight did not differ significantly among the groups. However, rabbits in the NPE300 group had significantly higher final body weight and weight gain compared to the PE0 group, while the PE300 group showed intermediate values. Feed intake showed numerical improvement in the NPE300 group, but the differences were not statistically significant. Both PE300 and NPE300 improved feed conversion ration compared to the PE0, however the difference was statistically significant only for PE300 compared to PE0.

### 3.3. Hemato-Biochemical and Immunological Responses

According to Table 3, rabbits that received NPE300 supplementation showed significantly higher values (*p* < 0.05) for white and red blood cell counts, packed corpuscular volume, hemoglobin concentration, lymphocyte percentage, monocytes, eosinophils, and phagocytic activity in comparison to the control group (PE0). The PE300 group generally showed values that were intermediate between the PE0 and NPE300. In terms of plasma biochemical indicators, both total protein and albumin levels were significantly elevated in the NPE300 group, whereas globulin levels did not vary significantly among treatments. Glucose and triglyceride levels were also significantly higher in the PE300 and NPE300 groups compared to PE0. Regarding immune markers, levels of immunoglobulin G and M were significantly increased in the NPE300 group, while immunoglobulin A showed no significant differences across treatments. For oxidative stress markers, malondialdehyde concentrations were significantly lower in rabbits supplemented with NPE300, while total antioxidant capacity did not show a significant difference among the three groups.

### 3.4. Intestinal Microbial Community

According to the data in Table 4, there were no significant differences among treatment groups in total bacterial count, Salmonella, or *E. coli* populations. However, the Clostridium count was significantly reduced (*p* < 0.05) in both PE300 and NPE300 groups compared to the control, with the lowest count observed in the NPE300 group. Additionally, the NPE300 group showed a significantly higher (*p* < 0.05) level of beneficial microflora compared to both the PE0 and PE300 groups.

### 3.5. Carcass Traits

Based on the data in Table 5, no significant differences were observed among the treatment groups for pre-slaughter weight, carcass weight without edible parts, total edible parts, or most organ weights. However, carcass weight with edible parts was significantly higher (*p* < 0.05) in the PE300 and NPE300 groups compared to the control. In terms of individual organs, fur and kidney weights were significantly greater (*p* < 0.05) in the NPE300 group compared to both PE0 and PE300. Cecum length was also significantly increased (*p* < 0.05) in NPE300 compared to PE0, while PE300 showed intermediate values. No significant differences were found in blood or stomach pH among the treatments.

## 4. Discussion

Pomegranate peel is an agro-industrial by-product with great potential for conversion from waste into valuable functional ingredients. As the most abundant and nutrient-rich part of the fruit, pomegranate peel contains high levels of phenolic compounds, which confer significant antioxidant and antimicrobial properties [21]. Dietary supplementation with pomegranate peel extract (PE) has been shown to promote growth, enhance feed conversion efficiency, and improve nutrient digestibility in growing rabbits. Additionally, PE exhibits notable antioxidant and antimicrobial effects, contributing to overall health and productivity [22].

To further enhance the biological efficacy and industrial applicability of these bioactive compounds, advanced delivery technologies such as nanoencapsulation have been increasingly explored [11]. These innovative approaches aim to improve the stability, bioavailability, and targeted delivery of phenolic compounds, thereby maximizing their beneficial effects on animal nutrition and health management.

In the present study, pomegranate peel extract (PE), whether nanoencapsulated or free, markedly enhanced its antimicrobial efficacy (Table 1). The superior inhibition zones observed for PE300 and NPE300 relative to PE0 likely reflect improved stability and sustained release of phenolics at the microbial interface (e.g., *E. coli*, *Salmonella*, *Vibrio*) when delivered in nanocapsules. Encapsulation within biopolymer matrices can protect phenolics from premature degradation and facilitate their interaction with bacterial cell walls, amplifying membrane disruption and oxidative damage to pathogens. This finding is consistent with Hashem et al. [5], who also demonstrated that nanoencapsulation of the phenolic compounds of PE may contribute to the improved viability in a synbiotic fula. Interestingly, phenolic compounds can selectively inhibit the growth of pathogenic bacteria without affecting the viability of probiotics and Gullon et al. [23] found that pomegranate juice, which is rich in ferulic, vanillic, and gallic acids, inhibited the growth of *Staphylococcus aureus*, *Pseudomonas aeruginosa*, and *Salmonella typhimurium*.

These antimicrobial actions were accompanied by notable shifts in the microbial profile of the small intestine (Table 4). Counts of *Clostridia* were markedly reduced, whereas beneficial bacterial populations increased under the PE300 and NPE300 treatments. This pattern is consistent with observations in growing rabbits supplemented with PE, where improvements in health indicators were linked to the selective suppression of pathogenic taxa while maintaining commensal communities. Furthermore, Sarıca and Ürkmez [24] reported that dietary inclusion of PE at 100 and 200 mg/kg was more effective than the control in improving total coliform levels in broilers. Collectively, these findings suggest that PE exhibits substantial antimicrobial potential and contributes to restoring microbial equilibrium within the gut [8].

These properties may directly reduce gastrointestinal infections, which in turn can lower the risk of diarrhea. Improved gut ecology under PE was accompanied by superior growth performance (Table 2). Rabbits receiving NPE300 and PE300 achieved higher weight gains and final body weights than PE0, consistent with better feed conversion and nutrient digestibility. Enhanced microbial profiles likely underlie this effect: increased beneficial microbes ferment polysaccharides to short-chain fatty acids (SCFAs), supplying additional energy to the host and stimulating enterocyte proliferation and villus height, thereby augmenting nutrient uptake. These effects may also be attributed to specific chemical compounds in the extracts. The PE provides animals with nutrients important for growth and contains high concentrations of syringic acid (SA), vanillic acid, and caffeic acid [25]. A broad range of beneficial actions for health has been attributed to phenolic acids, especially SA, which exhibit antioxidant, antimicrobial, anti-inflammatory, anti-endotoxic, neuroprotective, and hepatoprotective functions [26], supporting the view that pomegranate fruit is a medicinal and nutritional agent [21,27]. This improvement may be attributed to the proanthocyanidin in PE that may selectively enhance the growth of beneficial gut bacteria, suggesting a prebiotic activity for the extract [8], also, natural antioxidants which can protect the intestinal mucosa against oxidative damage and pathogens and limit peristaltic activity in digestive disorders preventing diarrhea [28,29].

At the systemic level, hematological and immunological parameters were also positively influenced by PE300 and NPE300 (Table 3). Elevated leukocyte counts, phagocytic activity, and immunoglobulins G and M indicate potentiation of both innate and adaptive immunity. Pomegranate peel is known to modulate signaling pathways in immune cells, reducing pro-inflammatory cytokine production while enhancing macrophage and lymphocyte function. Nanoencapsulation may further improve bioavailability of these bioactives, sustaining immunostimulation over the feeding period. The obtained results are supported by El-Sissi et al. [30], where a tendency for an increase in serum total protein and albumin levels was observed when PE300 and NPE300 were added to rabbit diets, reflecting enhanced immune function. These proteins play a crucial role in the immune system by activating lymphocytes and macrophages, regulating gene expression, and synthesizing specific proteins such as cytokines, antibodies, and cytotoxic substances. These findings are relatively consistent with those reported by Hagag et al. [31]. Concomitantly, oxidative stress markers were mitigated in NPE300-fed rabbits: malondialdehyde levels fell significantly, while total antioxidant capacity trended upward (Table 3). This dual action of PE—direct radical scavenging by polyphenols and upregulation of endogenous antioxidant enzymes has been documented in multiple species. Nano-formulation enhances these effects by protecting phenolics from early metabolism and targeting their release to absorptive sites in the gastrointestinal tract. The antioxidant and antiapoptotic capacities of pomegranate phytochemicals are triggered under chronic summer heat stress, confirming the earlier claim that the antioxidant potential of pomegranate has a broad and less specific effect [3]. The PE includes bioactive compounds that are abundant in the polyphenolic class of antioxidants, including tannins and flavonoids. In various pharmacological activities, such as anti-aging, anti-inflammatory, and anti-atherosclerotic activities, antioxidant activity has been suggested to play a vital function [32]. Antioxidant supplementation’s ability to prevent free radical damage has made it a popular treatment strategy for lowering disease risk [33].

Carcass and organ traits showed significant improvements with the inclusion of PE300 and NPE300 (Table 5). Specifically, carcass weight, including edible parts, as well as fur, kidney weights, and cecum length, were all increased, indicating enhanced overall growth performance and organ development. These findings are consistent with previous research; for instance, Imbabi et al. [34] reported that dietary supplementation with pomegranate extract (PE) positively affected the relative weights of various carcass cuts and internal organs, such as the saddle, thoracic neck, kidneys, and liver (*p* < 0.05). The antioxidant and antimicrobial properties of nano-encapsulated PE likely play a crucial role in this context by reducing subclinical infections and oxidative stress in tissues. This protective effect helps preserve muscle protein and connective tissue integrity, thereby supporting healthier organ morphology and function [35]. Overall, these results suggest that PE, especially in nano-encapsulated form, can effectively promote carcass quality and organ health through its bioactive effects.

## 5. Conclusions

Collectively, the findings of this study demonstrate a highly interconnected cascade of effects: nano-encapsulation enhances the delivery and stability of pomegranate phenolics, thereby amplifying their antimicrobial and antioxidant activities within the gastrointestinal tract. This improved functionality fosters a beneficial gut microbiota, which facilitates more efficient nutrient digestion and absorption. Consequently, systemic antioxidant defenses are strengthened and immune modulation is promoted, resulting in overall improvements in growth performance, health status, and carcass quality. This integrated mechanism underscores the potential of nano-encapsulated pomegranate extract as a powerful functional supplement in animal nutrition.

## Figures and Tables

**Table 1:** *In vitro* antimicrobial activities of pomegranate extract formulations against pathogenic microorganisms, expressed as inhibition zone (cm).

Pathogenic Microorganism	Treatment		
	PE0	PE300	NPE300
*IEscherichia coli ATCC 25922*	1.25 ± 0.03 ^b^	2.50 ± 0.30 ^a^	2.95 ± 0.10 ^a^
*Candida albicans ATCC 10231*	1.07 ± 0.06 ^c^	2.11 ± 0.10 ^b^	2.90 ± 0.20 ^a^
*Salmonella typhimurium ATCC 19430*	0.93 ± 0.03 ^c^	1.53 ± 0.03 ^b^	2.63 ± 0.20 ^a^
*Vibrio cholera NCTC 80211*	1.13 ± 0.06 ^c^	2.00 ± 0.01 ^b^	2.90 ± 0.20 ^a^
*Pseudomonas aeruginosa ATCC 27853*	1.13 ± 0.07 ^c^	1.8 ± 0.12 ^b^	2.23 ± 0.15 ^a^
*Bacillus subtills NCTC 80211*	1.23 ± 0.15 ^b^	1.67 ± 0.20 ^b^	2.33 ± 0.08 ^a^

^a,b,c^ Means in the same column followed by uncommon superscript letters are significantly different (*p* < 0.05). PE300 = non-encapsulated pomegranate extract (300 mg/L) with gum, NPE300 = Nano-encapsulated pomegranate extract (300 mg/L) using gum–gelatin-based nanocapsules.

**Table 2:** Growth performance of rabbits supplemented with non-encapsulated pomegranate-gum (PE300), pomegranate-gum nano-capsules (NPE300), or control treatment (PE0).

Variable	Treatment (*n* = 18/Group)			SEM	*p*-Value
	PE0	PE300	NPE300		
Initial body weight, g	899.68	904.67	898.00	4.55	0.842
Final body weight, g	1691.83 ^b^	1718.5 ^ab^	1843.83 ^a^	26.70	0.034
Weight gain, g	792.17 ^b^	813.83 ^ab^	945.82 ^a^	26.27	0.024
Feed intake, g	3977.50	4046.00	4159.80	48.89	0.325
Feed conversion ratio	5.02 ^a^	4.97 ^ab^	4.39 ^b^	0.152	0.025

^a,b^ Means in the same column followed by uncommon superscript letters are significantly different (*p* < 0.05). PE300 = non-encapsulated pomegranate extract (300 mg/L) with gum, NPE300 = Nano-encapsulated pomegranate extract (300 mg/L) using gum–gelatin-based nanocapsules.

**Table 3:** Hemato-biochemical and immunological attributes of rabbits supplemented with non-encapsulated pomegranate-gum (PE300), pomegranate-gum nano-capsules (NPE300), or control treatment (PE0).

Variable	Treatment (*n* = 8/Group)			SEM	*p*-Value
Hematology Parameters	PE0	PE300	NPE300		
White blood cells, 10^3/^mm^3^	22.15 ^c^	25.88 ^a^	27.36 ^a^	0.691	<0.0001
Red blood cells, 10^6/^mm^3^	4.99 ^b^	5.52 ^ab^	5.97 ^a^	0.158	0.020
Packed corpuscular volume, %	33.92 ^c^	35.51 ^b^	43.41 ^a^	2.77	<0.0001
Phagocytic activity	20.52 ^b^	21.89 ^ab^	23.03 ^a^	0.389	0.011
Lymphocytes, %	34.30 ^c^	35.01 ^b^	37.77 ^a^	0.727	0.009
Monocytes, %	12.19 ^b^	14.43 ^a^	16.06 ^a^	0.564	0.003
Eosinophil, %	12.22 ^b^	13.53 ^a^	13.77 ^a^	0.352	0.014
Heterophil, %	21.59	21.85	21.45	0.203	0.760
Hemoglobin, g/dL	12.25 ^c^	13.19 ^b^	14.24 ^a^	0.310	0.012
Plasma metabolites					
Total protein, g/dL	5.18 ^b^	5.76 ^b^	6.81 ^a^	0.248	0.007
Albumin, g/dL	2.75 ^b^	3.25 ^ab^	4.09 ^a^	0.243	0.004
Globulin, g/dL	2.43	2.51	2.72	0.158	0.769
Glucose, mg/dL	75.28 ^b^	82.37 ^a^	83.65 ^a^	0.401	0.012
Triglycerides, mg/L	100.22 ^b^	121.72 ^ab^	124.16 ^a^	0.494	<0.0001
Immunoglobulins, mg/dL					
Immunoglobulin G	994.32 ^b^	996.57 ^ab^	1011.65 ^a^	3.544	0.008
Immunoglobulin M	238.37 ^b^	251.87 ^ab^	259.969 ^a^	3.479	0.017
Immunoglobulin A	82.24	82.57	85.15	0.358	0.781
Antioxidant status					
Total Antioxidant capacity, mmol/L	1.69	1.89	2.00	0.126	0.125
Malondialdehyde, nmol/L	5.18 ^a^	4.89 ^a^	3.62 ^b^	0.270	0.021

^a,b,c^ Means in the same row followed by uncommon superscript letters are significantly different (*p* < 0.05). PE300 = non-encapsulated pomegranate extract (300 mg/L) with gum, NPE300 = nano-encapsulated pomegranate extract (300 mg/L) using gum–gelatin-based nanocapsules.

**Table 4:** Small-intestine microflora composition of rabbits supplemented with non-encapsulated pomegranate-gum (PE300), pomegranate-gum nano-capsules (NPE300), or control treatment (PE0) at 89 days of age.

Variable	Treatment (*n* = 8/Group)			SEM	*p*-Value
	PE0	PE300	NPE300		
Total bacteria Count, 10^6^ CFU/g	3.00	2.70	3.00	1.141	0.239
*Salmonela*, 10^5^ CFU/g	0.65	0.64	0.59	0.250	0.736
*E. coli*, 10^5^ CFU/g	1.83	2.15	2.17	0.123	0.473
*Clostridium*, 10^5^ CFU/g	1.68 ^a^	1.48 ^b^	1.33 ^b^	0.057	0.021 0.021
Micro flora, 10^5^ CFU/g	0.60 ^b^	0.64 ^b^	1.08 ^a^	0.089	0.030

^a,b^ Means in the same row followed by uncommon superscript letters are significantly different (*p* < 0.05). PE300 = non-encapsulated pomegranate extract (300 mg/L) with gum, NPE300 = nano-encapsulated pomegranate extract (300 mg/L) using gum–gelatin-based nanocapsules.

**Table 5:** Carcass traits of rabbits supplemented with non-encapsulated pomegranate-gum (PE300), pomegranate-gum nano-capsules (NPE300), or control treatment (PE0).

Variable	^1^ Treatment (*n* = 8/Group)			SEM	*p*-Value
	PE0	PE300	NPE300		
Pre-slaughter weight (g)	1750	1885	1921	38.42	0.160
^2^ Weight of the carcass with the edible (g)	1401 ^b^	1550 ^a^	1536.25 ^ab^	33.98	0.001
Weight of the carcass without the edible (g)	938.750	1072.50	1043.750	29.98	0.139
Weight the total of the edible (g)	468.75	477.50	518.75	14.08	0.190
Carcass Traits (g)					
Fur	295 ^b^	297.50 ^b^	332.50 ^a^	7.62	0.001
Kidney	12.88 ^b^	13.23 ^b^	15.23 ^a^	0.38	0.008
Heart	5.95	6.46	6.61	0.20	0.412
Lungs	10.87	11.01	12.22	0.72	0.724
Spleen	1.67	1.53	0.93	0.16	0.130
Intestine	97	117	115	5.29	0.242
Cecum Length, Cm	75.75 ^b^	96 ^ab^	105.75 ^a^	5.62	0.006
Blood PH	7.47	7.49	7.53	0.06	0.934
Stomach PH	6.06	5.85	5.97	0.05	0.290

^a,b^ Means in the same row with different superscript letter following them are significantly different (*p* < 0.05). ^1^ PE300 = non-encapsulated pomegranate extract (300 mg/L) with gum, NPE300 = Nano-encapsulated pomegranate extract (300 mg/L) using gum–gelatin-based nanocapsules. ^2^ Edible Giblets = kidneys, heart, lungs, spleen and the intestinal tract.

## Data Availability

Data of this study is confidential.
